# Effect of Milk and Water Kefir Grains on the Nutritional Profile and Antioxidant Capacity of Fermented Almond Milk

**DOI:** 10.3390/molecules30030698

**Published:** 2025-02-05

**Authors:** Chiara La Torre, Paolino Caputo, Alessia Fazio

**Affiliations:** 1 Department of Pharmacy, Health and Nutrition Sciences, University of Calabria, 87036 Arcavacata di Rende, Italy; chiara.latorre@unical.it; 2 Department of Chemistry and Chemical Technologies, University of Calabria, 87036 Arcavacata di Rende, Italy; paolino.caputo@unical.it

**Keywords:** water kefir grains, milk kefir grains, almond milk, microbial population, pH, fatty acid profile, phenolic content, antioxidant capacities

## Abstract

Today, the global trend toward plant-based beverages has grown for sustainability, health-related, lifestyle, and dietary reasons. Among them, drinks produced from almonds have been recognized as a concentrated nutrient source. Commercial almond milk was fermented under the same processing conditions using water and milk kefir grains to determine the starter culture leading to the beverage with the better nutritional profile. The resulting fermented beverages were investigated for protein, phenolic, and flavonoid content, fatty acid profile, and antioxidant activity, determined by DPPH, ABTS, and FRAP assays. Comparing the results, it was found that the almond beverage from milk kefir grains had the highest protein. The lipid profile of both beverages was characterized by a high content of monounsaturated fatty acids and a lower saturated fatty acid concentration compared to almond milk. Despite the higher phenolic content of the almond beverage from milk kefir grains, the ABTS and DPPH tests showed increased antioxidant activity in both fermented beverages, but with no significant difference between them, while the FRAP test showed a pronounced predominance of iron-reducing ability in the beverage from water kefir grains. The evidence from this study suggested that both types of grains can be used as starter cultures to enhance the nutritional and bioactive properties of almond milk.

## 1. Introduction

Nowadays, with the rise in the popularity of being ‘vegan’ and the ethical, environmental, and health concerns associated with dairy products [1], the global trend towards plant-based beverages produced mainly from nuts and cereals, such as soy, almonds, rice, oats, and coconut drinks, has increased [2,3]. The term “Plant-Based” is “used to describe a recent consumer trend of avoiding animal-based products and choosing plant-based alternatives instead” [4]. In particular, plant-based milk alternatives, otherwise known as PBMA, have been described as “liquids extracted by crushing plant material in water and designed by homogenization to be quite similar to cow’s milk in appearance, mouthfeel, taste, and shelf life so that they can be used for similar applications” [5]. On the basis of their vegetative sources, they have been classified as legume-, cereal-, pseudocereal-, seed,- and nut-based. Although some plant milk products contain low protein and calcium, plant milk substitutes are used to replace cow milk because of low allergy and intolerance issues, and lactose-free, cholesterol-free, and low-calorie diets [6]. Several plant-based milk alternatives, which have a long tradition in Eastern and Western cultures, are widely available on the market [3]. Among them, those produced from almonds have been recognized as a concentrated nutrient source, since almonds are rich in fatty acids, lipids, amino acids, proteins, carbohydrates, vitamins, minerals, and bioactive substances such as hydrolyzable tannins, proanthocyanidins, and flavonoids [7]. Additionally, almonds have a high K/Na ratio, and the available carbohydrates possess a low glycemic index [8]. In this context, almond drink stands out as a milk alternative with new functional features due to its nutritional content and health benefits. However, fermentation with lactic acid bacteria can improve the nutritional value of milk by increasing amino acids and vitamins, as well as therapeutic values, such as anti-microbial, antitumor, anti-carcinogenic, and immunomodulation activity [9], and degrade antinutritional compounds found in plants, such as tannins, saponins, phytic acid, α-galactosides, and trypsin inhibitors. This process enhances the beneficial quality of the product [1]. Studies have shown that almond drink is a suitable substrate for the growth of lactic acid bacteria (LAB) and kefir cultures [10,11]. They can enhance various functional properties, such as the release of phenolic compounds, conversion of glycoside isoflavones, and vitamin production [10], making the product an excellent functional drink. Among probiotic beverages, kefir is a currently well-recognized product whose production has increased considerably over the years, especially since the period of the COVID-19 pandemic. Kefir is a fermented beverage obtained by inoculating animal milk or any other sugary liquid matrix with milk kefir grains (MKGs) or water kefir grains (WKGs). Although similar, WKGs and MKGs show typical peculiarities regarding shape and color, as well as chemical and microbiological composition. It is precisely this latter aspect that is the most important variable that affects the overall features of the resulting beverage. Both MKGs and WKGs contain microorganisms in symbiotic relationships, namely LAB, yeasts, and acetic acid bacteria (AAB), but they were characterized by different ratios and genera of bacteria and yeasts. The prevalent bacteria in MKGs were *Lacticaseibacillus paracasei* subsp. *paracasei*, *Lactobacillus acidophilus*, *Lactobacillus delbrueckii* subsp. *lactis*, *Lactiplantibacillus plantarum* subsp. *Plantarum,* and *Lactobacillus kefiranofaciens* subsp. *kefiranofaciens* [12]. The LAB of WKGs belongs to the *Lactobacillus*, *Lactococcus*, *Leuconostoc*, *Pediococcus*, and *Streptococcus* species. The microbial composition of the grain inoculum used for the fermentation influences the microbial diversity and the characteristics of the fermentation process, providing unique properties to each fermented product [13]. According to the literature [14], there are some documents published between 2013 and 2022 that relate the keywords water kefir and plant-based milk [15,16,17,18] but, to the best of the author knowledge, only partial research has been undertaken concerning the fermentation of almond-based milk by milk kefir grains [19]. In this context, the aim of this study was to produce fermented beverages based on almond commercial milk alternatives by using both water (WKGs) and milk kefir grains (MKGs) under the same processing conditions and to evaluate the beverage chemical composition, physical chemical parameters, microbiological counts, and in vitro antioxidant activity by DPPH, ABTS, and FRAP tests. The results were compared to determine which type of grains led to fermented beverages with a better nutritional profile.

## 2. Results and Discussion

### 2.1. Water Content

The samples were freeze-dried to determine their water content. The data reported in Table 1 highlights that the starting milk, i.e., almond milk (AM), had the highest water content, if compared with the final beverages. In accordance with the data reported in the literature [12,20], there was a significant difference (** *p* < 0.01) between the moisture in the almond-fermented beverage with the water grains (AWK) and with the milk kefir grains (AMK). Also, the water content of the AMK and AWK significantly differed from the AM one (*** *p* < 0.001, *** *p* < 0.01, respectively). In fact, AWK had a lower viscosity and a semi-translucent appearance while AMK was a viscous, opaque dairy beverage.

### 2.2. Microbial Count

The microbiological quality of kefir was determined by counting colony-forming units (cfu) and monitoring the critical components of the kefir microflora, namely the total viable aerobic mesophilic bacteria, *Lactobacilli*, *Lactococci*, acetic acid bacteria, and yeast, using selective media for each group [21]. Table 2 shows the logarithmic counts (Log_10_ cfu/mL) of the total bacteria, *Lactobacilli* (LAB), *Lactococci*, acetic acid bacteria (AAB), and yeast determined in both kefir samples (AMK and AWK), as well as in both types of grains after 24 h of fermentation. Previous studies have investigated how microbial distribution within the grains can influence the population of bacteria and yeast in the fermentation medium during the process [22,23]. The results have shown that microorganisms located on the outer layers of the grains can migrate into the medium and ferment the sugars present, while those located in the inner layers contribute to the production of the exopolysaccharide matrices [13,24]. The microbial differences in milk kefir grains (MKGs) and water kefir grains (WKGs) resulted from the different initial composition of the two types of grains, which, in turn, depended on their geographical origin and biomass growth protocols, hygienic conditions, and production processes, such as the fermentation substrate, ingredients, and processing parameters [25]. *Lentilactobacillus kefiri*, *Lactobacillus kefiranofaciens* subsp. *kefiranofaciens*, *Lactobacillus kefiranofaciens* subsp. *Kefirgranum,* and *Lentilactobacillus parakefiri* were the characteristic bacteria in the MKGs [26]. *Lacticaseibacillus paracasei* subsp. *paracasei*, *Lentilactobacillus hilgardii*, *Liquorilactobacillus nagelii,* and *S. cerevisiae* were essential microorganisms for the fermentation of the WKGs [27]. Even though both types of kefir grains mainly contained LAB and yeast, there were differences in the species that affected final product characteristics. The microbial count showed that the species constituting the microbiota of both kefir grains and the corresponding fermented beverages were coincident, showing mainly differences in the relative abundance of these microorganisms. Total bacteria and AAB were significantly predominated in the WKGs (**** *p* < 0.0001), while LAB, *Lactococci,* and yeasts were significantly higher in the MKGs (**** *p* < 0.0001) after 24 h of fermentation. The absolute most prevalent microorganisms in the MKGs were *Lactococci* (4.57 ± 0.01 log_10_ cfu/g) while in the WKGs were AAB (4.30 ± 0.03 log_10_ cfu/g).

The corresponding fermented beverages (AMK and AWK) presented a different relative abundance of the microorganisms, with *Lactococci* being the predominant species in the AMK and AWK (4.60 ± 0.01 log_10_ cfu/g and 3.48 ± 0.01 log_10_ cfu/g, respectively). In both fermented products, AMK and AWK, the microbial counts of all species were lower than in the corresponding grains except for *Lactococci* and yeasts, which populated both the AMK and the MKGs with the same concentration. LAB was missed in both beverages. The microbial analysis confirmed the absence of molds. The AWK did not have higher counts of yeast counts than the AMK one, which was expected, since yeasts are usually in higher concentration in MKGs [28].

Our results about the microbial population in the fermented beverages were found to be lower than those reported in the literature. The study performed by Ustaoğlu-Gencgonul et al. [29] reported that almond kefir from WKGs contained 8.3 log cfu/mL of *Lactobacillus* spp., 7.73 log cfu/mL of *Lactococcus* spp., and 5.36 log cfu/mL of yeast. Comak Gocer and Koptagel [30] found that almond beverage obtained by inoculating milk from raw almonds using WKGs for 24 h had a population of total aerobic mesophilic bacteria, *Lactococci*, *Lactobacilli*, AAB, and yeast equal to 8.81 ± 0.31, 9.26 ± 0.34, 8.76 ± 0.24, 8.82 ± 0.25, and 4.20 ± 1.86 log cfu/g, respectively. The higher values of the microbial counts in both cited studies compared to our findings were probably due to the better quality of the starting milk, having been homemade as an aqueous extract of raw almonds, which was a good source for fermentation of the WKGs. To the best of our knowledge, there was no data in the literature regarding the microbiological composition of almond kefir obtained by fermentation with MKGs.

### 2.3. pH and Total Acidity

The pH value of the AM was 7.68 ± 0.16 and it dropped to 4.71 ± 0.02 after 24 h of fermentation by MKGs but it was further reduced if the starter culture used was WKGs (Table 3). The pH values of the AMK and AWK significantly differed between them (** *p* < 0.01) and compared to the AM (**** *p* < 0.0001). The significant difference between the pH values of the two types of fermented beverages was probably related to the nature of the sugars (4.1 g/100 mL) contained in the AM. According to the pH values, the titratable acidity of the AWK was significantly higher than the AMK and AM ones (**** *p* < 0.0001) (Table 3). Also, the titratable acidity of the AMK was more significant than the AM one (*** *p* < 0.001). The results were expected since the almond milk did not contain the lactose that the bacteria in milk kefir cultures usually convert into lactic acid, causing the pH drop, and the main sugar was sucrose (4.1 g/100 mL). On the contrary, yeasts in AKGs quickly broke down sucrose into monosaccharides (glucose and fructose), which were then consumed by LAB and AAB, thereby increasing the production of lactic acid and acetic acid during the fermentation process. Rios [31] evaluated the pH of five fermented beverages with MKGs based on vegetable extract from rice, brown rice, coconuts, cashew nuts, and Brazil nuts. The findings led to the conclusion that the Brazil nut-based beverage did not decrease pH, which may be explained by the composition of the plant extract, which might have directly affected the metabolism of the microorganisms. Due to vast differences in microbiological composition, MKGs and WKGs were usually not interchangeable. MKGs worked better in milk or whey-based medium and sometimes could be grown in plant-based milk. WKGs required vegetable/fruit/cereal-based solutions with sufficient fermentable sucrose or fructose. Our results corroborated that the plant-based milk created a more favorable environment for the growth of WKGs than for MKGs. However, the titratable acidity values in fermented drinks prepared from plant-based milk, such as almond milk, were lower than those found in fermented cow milk [32] due to their different buffering capacities. Previous studies have found that buffering capacity was influenced by protein and amino acid content, acids, and base groups, such as salts and organic acids [33]. Cow milk has a high buffering capacity due to the presence of organic acids, salt, and proteins [34]. In contrast, almond-based milk has a lower buffering capacity since the high concentration of fat hinders the reaction between the titrant and protein, which are lower than in cow milk ones [35]. Our results for the pH and total acidity values of the AWK were in agreement with the data reported in the literature, where the pH and total acidity values of the almond-fermented beverage ranged from 4.0 to 4.80 and from 0.18% to 0.26%, respectively [12,30].

### 2.4. Kefir Grains Growth

The biomass growth of both MKGs and WKGs, as a result of the fermentation process, was reported as a percentage (%, *w*/*w*). The MKGs’ wet mass remained unchanged during the fermentation process. In contrast, WKGs significantly grow from their initial weight by 84% (**** *p* < 0.0001). Several factors usually influence grain biomass growth, including incubation time, temperature, and especially growing medium composition. Rodríguez-Figueroa et al. [36] investigated the effect of different substrates on the kefir grain biomass increase. Goat milk was the substrate associated with the highest kefir grain biomass productivity. On the other hand, the presence of brown sugar significantly limited the biomass increase [36]. Grain biomass increment was associated with the synthesis of the exopolysaccharide by microorganisms coexisting in the grain. The main component of the WKGs’ matrix was a glucan-type homopolysaccharide containing mainly α-1,6 linked glucosyl units and a few ramifications formed by α-1,3 glycosidic linkages [37]. *Lacticaseibacillus casei*, *Liquorilactobacillus hordei,* and *Lentilactobacillus hilgardii* were considered the main ones responsible for generating the dextran structure in the WK grains [38]. Martinez-Torres et al. [39] reported that WKGs do not grow in milk since *L. hilgardii* did not metabolize lactose, but they did in plant-based milks that were rich in sucrose. The main bacterial polysaccharide specific to the MK grains was known as “kefiran”, which has been described as a water-soluble glucogalactan that contains approximately equal amounts of glucose and galactose with 127 hexose units [40]. Studies have indicated that the main bacterial species responsible for producing the exopolysaccharide in MKGs were *Lactobacillus kefiranofaciens* subsp. *kefiranofaciens*, *Lentilactobacillus kefiri* and *Lactobacillus kefiranofaciens* subsp. *kefirgranum.* Ghasemlou et al. [41] found that lactose gave the highest biomass of kefir grains and the highest kefiran yield of 4.3% based on the kefir grain biomass, followed by sucrose. Nevertheless, it should be noted that *Lactobacillus kefiranofaciens* subsp. *kefiranofaciens* needed nitrogen sources to grow and produce kefiran, so it lost its effectiveness in almond milk, which is a poor-protein medium, explaining the lack of biomass growth in MKGs [42].

### 2.5. Total Protein Content

The protein composition of kefir generally varies as it depends on the source of milk, the components of the grains or cultures, and the process of kefir fermentation [43]. In the present study, it was the different nature of the kefir grains that determined the difference in protein concentration (Table 4). Almond milk contained 0.4 mg/100 mL of protein, but the fermentation with MKGs led to a significant increase in the protein of 62.7% compared to the starting amount (*** *p* < 0.001) and even higher than the WKGs one (** *p* < 0.01). Hew et al. reported that the protein content of almond milk, prepared from raw almonds and inoculated by MKGs, was 5.69%, which was higher than our result. This difference was probably related to the different composition of the starting milks [44]. 

Regarding our results, the significant difference in protein content in the AMK and AWK was probably attributable to the different proteolytic activity of the microorganisms that characterized the composition of the MKGs and WKGs [21]. Among the various microorganisms living in the grains, the most important proteolysers were LAB. They contained a cell envelope proteinase (CEP) that hydrolyzed proteins into oligopeptides, which were further degraded into shorter peptides and amino acids by intracellular peptidases. The higher LAB count in the MKGs than in the AKGs would explain the higher concentration of proteins in the AMK than in the AWK.

### 2.6. Fatty Acid Content

Table 5 shows the values of saturated, monounsaturated, and polyunsaturated fatty acids in the AM, AMK, and AWK. Saturated fatty acids, such as palmitic, heptadecanoic, and stearic acids, were found in all milk samples.

In the AM, among the monounsaturated fatty acids, C4:0 was the most abundant (120.83 ± 2.44 mg/g), while C18:1n-9 was the predominant monounsaturated fatty acid (188.77 ± 2.20 mg/g), and C18:2n-6 (34.37 ± 0.48 mg/g) was the most representative polyunsaturated fatty acid. Karimi et al. [45] indicated that 66% of almond fats were monounsaturated, 26% polyunsaturated, and 8% were saturated fats. In both the AMK and AWK beverages, methyl butyrate (C4:0) significantly decreased by 47.64% (63.27 ± 9.32 mg/g) and 35.72% (77.67 ± 0.40 mg/g), respectively, during fermentation (**** *p* < 0.0001), whereas C18:1n-9 increased by about 1.46-fold in both fermented beverages (**** *p* < 0.0001). C18:2n-6, from the initial value, reached a concentration of 48.91 ± 0.68 mg/g and 71.32 ± 2.69 mg/g in the AMK and AWK, respectively (**** *p* < 0.0001). Methyl linoleate, which was absent in the AM and AMK, formed during the fermentation process in the AWK (**** *p* < 0.0001).

In both fermented beverages, the MUFA component prevailed over the SFAs and PUFAs and was higher than in the AM (**** *p* < 0.0001), while the AWK had a major concentration of PUFAs with respect to both the AM (*** *p* < 0.001) and AMK (** *p* < 0.01). Monounsaturated fatty acids have been shown to have anti-carcinogenic benefits based on data gained in experimental animals and human cell culture investigations [46]. The population of LAB in kefir grains, endowed with lipase activity, was responsible for the degradation of complex lipids into free fatty acids, which led to a different quantitative and qualitative fatty acid profile in the AMK and AWK [47]. Our results regarding the predominance of MUFAs in the AMK agreed with those reported by Comak Gocer and Koptagel, apart from the content of PUFAs that exceeded SFAs [30].

### 2.7. Total Phenolic Content (TPC) and Total Flavonoid Content (TFC)

Table 6 reports the changes in the TPC and TFC of the fermented AMK and AWK compared to the AM. It had a TPC of 165.43 ± 0.57 µg GAE/mL, which was within the range of values reported in the literature [48]. Manzoor et al. [49] reported a TPC value of 702.2 μg/g for thermosonicated almond drinks, while Ceylan [50] found TPC values in almond drinks ranging from 102 mg/kg to 553 mg/kg. This wide range of results could be related to the different mass fractions of the TPC in the almonds used as raw material and the different preparation methods of the almond drinks in the mentioned studies. The TPC value significantly increased by 2.2-fold after fermentation with MKGs and 1.7-fold in the AWK, compared to the initial value (**** *p* < 0.0001). Similarly, the total flavonoid content was significantly higher in the AMK than it was at the initial stage (** *p* < 0.0001), and also significantly higher in the AWK (**** *p* < 0.0001), which, in turn, significantly differed from the AM (** *p* < 0.01).

The increase in the TPC and TFC in both fermented almond milks may be related to the metabolic activity of microorganisms, such as some lactic acid bacteria (LAB), which were strain- or species-specific [51]. They were able to bioconvert bound phenolic compounds from their linked or conjugated forms to their free ones (aglycones). This mechanism was based on the breakdown of the bonds with the plant cell wall components by the activities of enzymes (such as β-glucosidase, decarboxylases, esterases, hydrolases, and reductases) as well as the metabolic activity of the fermenting microorganisms [52,53]. In addition, the progress in the acidity of the samples presumably induced the delinking of some phenolic compounds [54,55]. Phenolic compounds had higher bioavailability in their free form, and the released aglycones had the potential to increase polyphenol concentration. The levels of enzymatic activity were found to vary among the different types of grains during milk fermentation [56]. In addition, the prevalence of LAB in the MKGs could justify their greater metabolic activity towards source milk proteins than the LAB in the WKGs, where they were lower. 

### 2.8. HPLC-DAD Analyses of Biophenols

HPLC-DAD analyses confirmed the presence of eight biophenolics in the AM, AMK, and AWK (Table 7). The identified compounds were gallic, chlorogenic, vanillic, *p*-coumaric, ferulic, ellagic acids, catechin, and quercetin.

The main phenolics identified in the AM were vanillic (6.76 ± 0.31 µg/g), *p*-coumaric acids (5.83 ± 1.45 µg/g), and catechin (6.40 ± 0.03 µg/g). Though several phenolic compounds, including phenolic acids, flavonoids, and, to a lesser extent, isoflavones, stilbenes, and lignans, have been characterized in almonds [57,58], Grainger et al. [59] only detected phenolic acids and hydroxybenzaldehyde, according to our results. After fermentation, the total amount of the identified polyphenols (18.99 ± 1.79 µg/g) in the AM increased to 34.36 ± 0.1 µg/g in the AMK and even more so in the AWK (39.68 ± 0.41 µg/g). More specifically, *p*-coumaric acid doubled in the AWK (10.81 ± 0.15 µg/g) from the initial content of 5.83 ± 1.45 µg/g due to the fermentation process, but, even more interestingly, biocompounds, such as ferulic and chlorogenic acids, as well as quercetin, were not present in the AM, but they subsequently formed in the AMK and AWK. Gallic acid was found at between 1.32 ± 0.01 and 1.35  ± 0.01 µg/g. Gallic acid is known for its high bioavailability and antioxidant effect among hydroxybenzoic acid derivatives [60]. Chlorogenic acid reached levels of around 2.5  ± 0.01 µg/g in both fermented drinks, and quercetin was the major phenolic component in the AMK and AWK (8.82 ± 0.01 µg/g). In accordance with the trend of TPC values increasing in both the AMK and AWK after 24 h of fermentation, the rise of individual phenolic compound content was the consequence of the enzymatic activity of microorganisms during fermentation that degraded the complex, unidentified phenols of the starting milk into simpler structures that could rearrange into phenolic acids and aglycones. These simpler structures were very good at fighting free radicals [11,61,62].

### 2.9. Antioxidant Capacities of AM, AMK, and AWK

The antioxidant activities of all samples were determined by performing three different in vitro assays: ABTS and DPPH assays, as well as the FRAP test.

#### 2.9.1. DPPH Assay

The TEAC values of the AM, AMK, and AWK against DPPH, as well as the corresponding EC_50_ values, are reported in Table 8. The TEAC values of the AM in the range of 33.33–333.33 µg/mL concentrations remained at high values between 182.78 ± 5.55 and 221.35 ± 1.51 µg TE/mL. These values were higher than those reported by Jemaa et al. [63] and even by Balbino et al. for almond-based dairy-free milk alternative formulation fortified with myrtle, bay leaf, and fennel extracts [64]. In the study by Lipan et al. [65], the determined antioxidant activity of the almond drink was 47 μmol/L, which was significantly lower than the value determined in this study. Nevertheless, our findings were lower than the value of 1310 μmol/L determined by Plank et al. [66]. The differences in antioxidant activity reported in the literature could be attributed to variations in the raw materials used and the differing processing conditions employed in the preparation of almond drinks.

The fermentation process by the MKGs resulted in an increase in antioxidant activity in the same concentration range between 184.21 ± 5.55 and 231.36 ± 0.50 µg TE/mL. Slightly higher values were found in the AWK (198.50 ± 2.52–232.78 ± 1.51 µg TE/mL). The EC_50_ values for the AM, AMK, and AWK were calculated in order to establish which of the two fermented beverages had greater antioxidant capacity against the DPPH radical. 

The lowest EC_50_ value was found for the AWK (3.91 ± 0.58 µg/mL), which was significantly higher than the value for the AM (**** *p* < 0.0001). However, there was no significant difference between the EC_50_ values of the AWK and AMK (4.68 ± 0.66 µg/mL).

#### 2.9.2. ABTS Assay

Table 9 shows the TEAC values of the AM, AMK, and AWK against the cationic radical ABTS•^+^ and the corresponding EC_50_ values.

The ABTS free radical scavenging activities of the AMK and AWK samples were not found to be significantly different. Although the TE values were not so dissimilar between the milk sample and the fermented beverages, the calculation of the EC_50_ values made it possible to establish that the fermentation process resulted in a significant increase in antioxidant properties compared to the initial values (**** *p* < 0.0001), similar to what was observed for the DPPH test.

#### 2.9.3. FRAP Test

The FRAP values of all samples (Table 10), including the positive control (BHT), at a concentration of 3.3 mg/mL, significantly differed from each other (**** *p* < 0.0001) highlighting that WKGs presented greater FRAP activity than MKGs. The absolute highest FRAP value was found for the AWK (500.09 ± 1.93 μM/mL FeSO_4_).

Data collected by performing three assay tests in order to determine the antioxidant capacity of the kefir samples compared to the almond milk one highlighted that the ABTS and DPPH free radical scavenging activities of the AMK and AWK increased during the fermentation process, but the corresponding values were not significantly different, as demonstrated in Table 8 and Table 9, unlike the corresponding total phenolic contents, which were significantly different (**** *p* < 0.0001). In contrast, the ferric reducing capacity of the AWK was highly significant compared to the AMK one, although it did not reflect the trend of the TPC, increasing more in the AMK than in the AWK. Therefore, not all antioxidant activity analysis methods may yield similar results; the electron-donating abilities of AWK’s antioxidant compounds in the FRAP method surpassed their hydrogen-donating abilities in the DPPH method. Since the phenolic content increased as fermentation proceeded, but in a manner that did not correlate with the results of the DPPH, ABTS, and FRAP tests, these findings taken together suggested that phenolic compounds were a major contributor to antioxidant capacity in these beverages, but this was also presumably due to nonphenolics. These results were emphasized as potentially associated with the metabolic activity of different microorganisms in grains that released the enzymes responsible for breaking down complex structures into smaller compounds that could exert antioxidant activity [67].

### 2.10. Rheological Analysis

**Figure 1:**
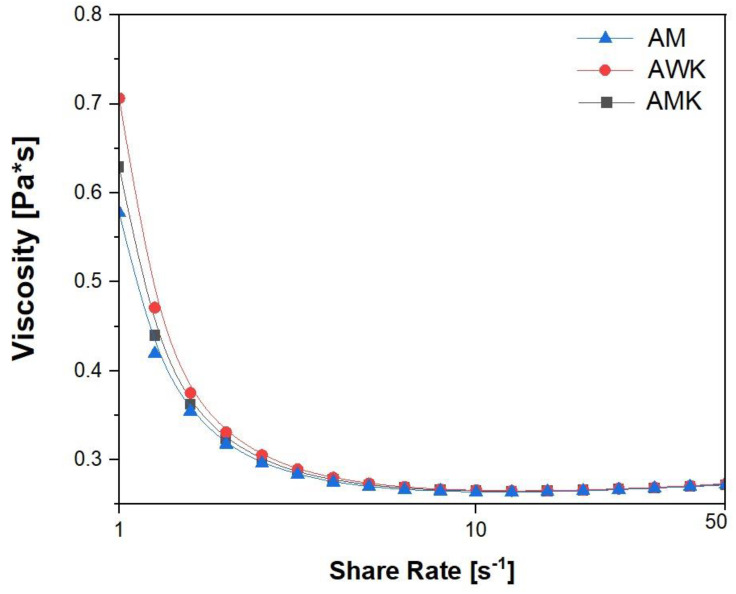
The flow behavior of the AM, AMK, and AWK.

The flow curves of all samples, at the studied concentration of 3.0 wt% in the shear rate 3.0–50 s^−1^ range, are plotted in Figure 1. The samples showed a non-Newtonian pseudoplastic behavior at a low shear rate. At a low shear rate (1.0 s^−1^), the AM exhibited the lowest viscosity value compared with the AWK and AMK ones. The flow curves became almost overlapped at a high shear rate (25–50 s^−1^), where the viscosity was around 0.27 Pa**^.^**s. All values of apparent viscosity as a function of the shear rate are listed in Table 11. The viscosity trends of all samples could be divided into two regions. The first region was within the 1.0–10 s^−1^ shear rate range, where the apparent viscosity of all the samples decreased and the second one was within 10–50 s^−1^, where the apparent viscosity of the samples dropped below 0.30 Pa**^.^**s. In the first region, the slope of the AWK profile was higher than in the other ones [30]. The flow curves showed that the examined kefirs were shear-thinning materials. This type of rheological behavior was typical for the three-dimensional structure samples being destroyed under the shear forces [68]. The decrease in apparent viscosity of the kefir samples during storage was attributed to a decrease in the amount of exopolysaccharide produced due to a drop in the LAB population being responsible for exopolysaccharide synthesis [69]. In addition, the viscosity of the kefir may decrease due to the weakening of its three-dimensional structure caused by bacterial proteolytic degradation. Furthermore, hydrolysis of exopolysaccharides by glycohydrolase enzymes from bacteria caused a decrease in viscosity. The acceptability of pseudo-plasticity was higher in the fermented beverages. Comak Gocer et Koptagel reported that the kefir samples made from almonds had an apparent viscosity of 0.083 ± 0.011 Pa**^.^**s at a shear rate of 50 s^−1^, which was lower than our findings [30].

Data reported in Table 11 shows that at the lowest shear rate (1.0 s^−1^), the AWK had the highest viscosity value (0.706 Pa·s). The trend of the apparent viscosity values indicated shear-thinning behavior that was expected in liquid fermented milk due to the loss of physical interactions between proteins caused by weak electrostatic and hydrophobic bonding [70].

### 2.11. Scanning Electron Microscopy

**Figure 2:**
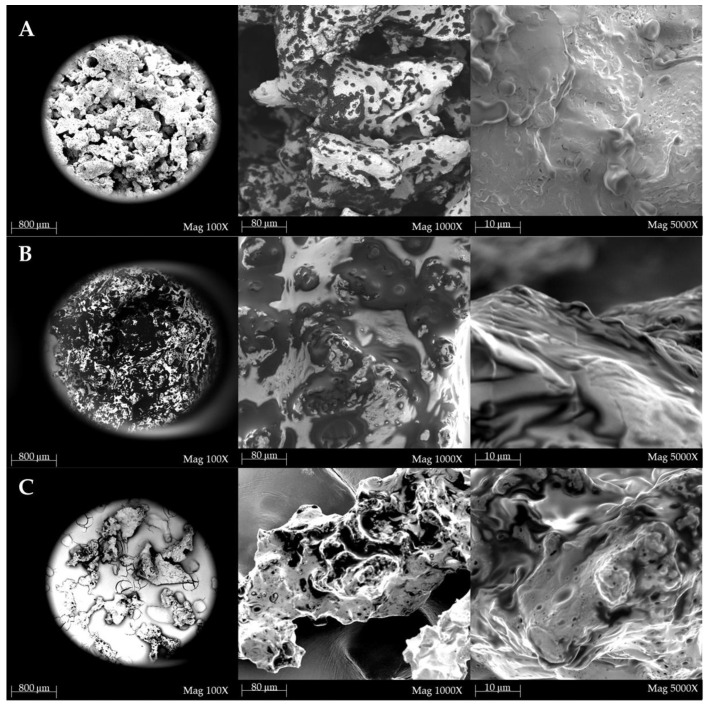
Scanning electron images of the AM (**A**), AMK (**B**), and AWK (**C**). The magnifications were equal to 100×, 1000×, and 5000×, with a scale of 800, 80, and 10 µm, respectively.

Figure 2 shows the micrographs obtained by Scanning Electron Microscope (SEM, Thermo Fisher Scientific, Hillsboro, OR, USA) of the sample powder particles produced by freeze-drying.

All samples showed a whitish background that can probably be attributed to substances with a higher atomic weight than the rest of the material (e.g., calcium). Particularly in the 1000× magnifications of all samples, dark spots were observed, which were probably to be attributed to organic substances. In the samples of AMK and AWK, such dark spots did not have a well-defined shape and, therefore, were to be ascribed to an amorphous structure, while in the AM they presented a more defined shape, in some cases round, suggesting that we were in the presence of an incipit of crystallization. Ice crystals formed due to the low temperatures and sublimated under reduced pressure, forming dry and porous products [71]. Almeida et al. [71] also observed porous surfaces, irregularly shaped, and disordered, highlighting different morphologies between the spray-dried kefir microparticles and the lyophilized ones. The three samples had different degrees of porosity: the AWK exhibited more porosity than the other two samples, evenly distributed and with the presence of pores, that, in some cases, were large, while the AMK had low porosity, and the AM exhibited an unevenly distributed microporosity.

### 2.12. Pearson Correlation

Pearson correlations between the TPC, TFC, and antioxidant capacity by ABTS, DPPH, and FRAP assays of the samples (AM, AMK, and AWK) are reported in Table 12. The results showed that in the AM, the TPC was positively correlated with the TFC and ABTS, and DPPH with FRAP, while in both the AMK and AWK, the TPC was positively correlated with the TFC, DPPH, and ABTS. Antioxidant capacities measured by ABTS and DPPH assays reported a strong positive correlation with a Pearson correlation coefficient of r = 1 in the AMK and r = 0.86 in the AWK. The FRAP values were negatively correlated with the TPC (r = -0.96 n AWK; r = −0.82 n AMK). 

## 3. Materials and Methods

### 3.1. Material and Reagents

Almond milk (Valsoia Spa, Bologna, Italy) was acquired from a local market. The nutritional values of the milk, stated on the label, per 100 mL, were as follows: fat, 1.1 g of which was saturated, fatty acids 0.2 g, carbohydrates, 4.1 g of which were sugars 3.9 g, fiber 0.1 g, protein 0.4 g, and salt 0.05 g. Milk and water kefir grains were provided from Kefiralia (Arrasate, Gipuzkoa, Spain). Milk kefir grains contained *Lactococcus* spp., *Leuconostoc* spp., *Lactobacillus* spp., and yeast [21]. The initial microbial composition of the water grains consisted of 7–8 log cfu/g of lactic acid bacteria (LAB), mainly *Lactobacillus* spp., *Lactococcus* spp., *Leuconostoc* spp., and *Streptococcus* spp., of acetic acid bacteria (AAB), especially *Acetobacter Lovaniensis* and 6–7 log cfu/g of yeasts, commonly *Saccharomyces* sp., *Kluyveromyces* sp., *Pichia* sp., and *Candida* sp.. All solvents (ACS grade) were from Carlo Erba Reagent (Milan, Italy), and all reagents were obtained from Sigma Aldrich (Milan, Italy). All chemicals used for chromatographic analyses were of chromatographic purity.

### 3.2. Kefir Grain Activation

Fresh milk kefir grains were activated through a series of consecutive prefermentations. These were carried out at room temperature in a glass vessel containing 500 mL of pasteurized cow milk and covered with a muslin cloth in order to allow for aerobic fermentation conditions. The grains (100 g) were poured into the jar and gently mixed with a spoon. Every day, the grains were filtered through a plastic sieve and the milk was discarded and replaced by fresh milk [72]. The activation took 7 days.

Water kefir grains were activated through a series of three consecutive water kefir prefermentations, which were performed in a glass container covered with a breathable cloth. The grains (100 g) were added to 1 L of demineralized water, supplemented with 100 g of sucrose, and fermented at room temperature for 7 days. Every 48 h, a backslopping practice was applied, whereby the water kefir grains were separated from the water kefir liquor by sieving and recultivated in a fresh medium under the same conditions as mentioned above. 

### 3.3. Fermentation Process

Both activated grains (10 g, 10% *w*/*v*) were separately cultivated into almond milk (100 mL) for 24 h at room temperature. At the end of the process, the fermented milk was separated from the grains by a plastic sieve, frozen at −20 °C overnight, and freeze-dried (Telstar LyoQuest, Barcelona, Spain). The obtained powders were analyzed to determine their water, protein, phenolic and flavonoid content, and fatty acid profile, as well as their antioxidant activity, before and after fermentation [73].

### 3.4. Determination of Water Content

The total water content was determined gravimetrically by performing the difference between the weight of the fermentation product recovered after the separation of the grains and that of the powdered product at the end of freeze-drying.

### 3.5. Microbiological Analysis

At the end of fermentation, both the kefir grains and the final beverage were analyzed to evaluate their microbial content [21]. Ten grams of the freshly cultured grains and kefir were separately diluted (1:10 m/V) with a ringer solution (LAB M Limited, Lancashire, UK). Samples containing the grains were homogenized using a stomacher (Interscience BugMixer® 400 P, Saint Nom, France) for 15 min at maximum speed. Then, all samples were diluted from 10^−1^ to 10^−5^, and 0.1 mL of the proper dilutions were spread in triplicate onto plate using specific solid media: Man Rogosa Sharpe (MRS) agar was used as medium for *Lactobacilli*, M17 agar for *Mesophilic cocci*, GYP medium for acetic acid bacteria (AAB), PDA medium (potato dextrose agar) for yeasts and molds, and PCA medium (plate count agar) for total bacteria. The plates were then incubated at 30 °C under aerobic conditions (AAB, *t* = 48 h; yeast, molds, and total bacteria *t* = 72 h) or 37 °C for 5 days (*Lactococci* and *Mesophilic cocci*). After incubation, the colonies of each type of microorganism were counted, and the viable bacterial cell counts were calculated considering the dilution factors. The counting results were expressed as the means of the logarithm to base 10 of colony-forming units (log_10_ cfu) per gram of sample ± standard deviations.

### 3.6. Evaluation of pH and Total Acidity

The pH values before and after fermentation were determined by a pH-meter electrode (Hanna Instruments, Padua, Italy). After calibration, it was dipped into the samples at 25 °C. 

The titratable acidity of the kefir samples was determined using the AOAC titration method [74]. It is evaluated by titrating a known volume of milk with a standard alkali to the point of the phenolphthalein indicator. The titratable acidity test measured the amount of alkali that was required to change the pH of the milk from its initial value of about 6.6 to 6.8, up to the pH at the color change of phenolphthalein added to milk to indicate its endpoint (pH 8.3). In fact, this method measured the buffering capacity of milk and not the true acidity. Distilled water (40 mL) and 3 drops of phenolphthalein (1% *v/v* in 95% ethanol) were added to the milk sample (20 mL). The mixture was titrated with 0.1 M NaOH until the first color change (pink) persisted for 30 s, and then the final volume of 0.1 M NaOH was noted. The titratable acidity (TA) value was expressed as the % of lactic acid.

### 3.7. Kefir Grain Growth

Kefir grain biomass increase was determined gravimetrically. The grains from the fermented process were aseptically separated from the fermented milk by using a plastic sieve. Then, they were washed with water, centrifuged (5000 rpm, 10 min), and transferred into a Petri dish in which sterilized paper towels were placed to remove excess water. After the paper towels were removed, the kefir grains were weighed using an analytical balance (Discovery Ohaus, Parsippany, NJ, USA). The biomass growth was calculated using the following equation:

Biomass growth (%) = [grain weight (final) − grain weight(initial)/grain weight (initial)] × 100

### 3.8. Protein Content Determination

The Kjeldahl method is an official method that allows for the determination of the nitrogen content in organic and inorganic samples. It is used to evaluate the protein content of the samples because it is approved by Codex Alimentarius as the standard for quantifying milk proteins [75]. The Kjeldahl procedure involved three major steps: digestion, distillation, and titration. During the digestion procedure, all nitrogen bonds in the sample (5 mL) were broken due to the use of a high temperature (430 °C) and an acid, such as H_2_SO_4_ (98%, 10 mL), and all of the organic nitrogen was converted into ammonium ions (NH_4_^+^). K_2_SO_4_ (3 g) and the catalyst CuSO_4_·5H_2_O (300 mg) were added in order to increase the boiling point of sulfuric acid and the speed and efficiency of the digestion procedure, respectively. After digestion was completed, the ammonium sulfate was allowed to cool to room temperature, then diluted and heated after the addition of 30% NaOH (60 mL), which converted the ammonium sulfate to ammonia. It was distilled off and trapped in a receiving flask containing an excess of hydrochloric acid, forming ammonium chlorate. The residual hydrochloric acid was then titrated using 0.1 N NaOH with the use of a suitable end-point indicator (methyl orange) that highlighted a color change from pink to yellow. To estimate the total nitrogen content of the samples, a nitrogen-to-protein conversion factor of 6.38 was applied, based on purified acid casein typically containing 15.67% nitrogen [76].

### 3.9. Fatty Acid Methyl Esters Preparation 

In order to determine the fatty acid profile of the milk, samples were derivatized by transesterification into fatty acid methyl esters (FAMEs) [21].

An amount of 0.2 mL of derivatization reagent (acetyl chloride) was slowly added to a 10-mL glass vial that contained the freeze-dried sample (100 mg), solubilized in 2 mL of methanol–benzene 4:1 (*v/v*), and the vial was tightly sealed with a Teflon lined cap. The methylation reaction was conducted by incubation at 100 °C for 1 h under stirring. After cooling to room temperature, a 6% K_2_CO_3_ solution (5 mL) was added to each vial, and the mixture was centrifuged. Benzene upper phase (0.1 mL) was transferred into a vial and diluted with hexane up to 1 mL before gas chromatographic analyses.

### 3.10. GC Analysis of FAMEs

The separation of FAMEs was achieved by a fused-silica capillary column (30 m × 0.32 mm id × 0.25 µm, Supelcowax™ 10, Milan, Italy) with a constant flow of 1.2 mL min^−1^ helium as a carrier gas, a linear velocity (u) of 37.5 cm/s, using the following oven temperature program: an initial temperature of 60 °C, held for 5 min, raised by 16 °C min^−1^ to 185 °C, and maintained for 12 min, and subsequently increased by 20 °C min^−1^ to 235 °C and held for 14 min. The injection inlet temperature was 235 °C, and the injection volume was 1 μL [21]. The detection of FAMEs was performed by comparison with the GC analysis under the same temperature program conditions as the standards. The absolute quantization of each FA was carried out by a four-point calibration curve in the range of 0.1–1 mg/mL for each standard. Fatty acid contents in AM, AMK, and AWK were reported as average values ± standard deviation and expressed as mg/g.

### 3.11. Total Phenolic Content (TPC)

The total phenolic content was evaluated using the Folin-Ciocâlteu colorimetric method, according to the methodology described by La Torre et al. [77], performing each experiment in triplicate. A four-point calibration curve (y = 0.0874x + 0.0207, R^2^ = 0.998) using the gallic acid standard from 10 to 500 μg/mL was used to quantify the total phenolic content. The results, expressed as the mean value of three different replicates ± standard deviation (SD), were reported as micrograms of gallic acid equivalents per mL of the sample (μg GAE/mL).

### 3.12. Total Flavonoid Content (TFC)

The total flavonoid content (TFC) was evaluated by a colorimetric assay method [78]. An amount of 0.3 mL of the sample (100 mg/mL DMSO) was solubilized in MeOH (0.9 mL) and added with a 10% aluminum chloride solution (0.06 mL), sodium acetate solution (1 M, 0.06 mL), and distilled water (1.68 mL). After incubation in the dark for 30 min under stirring, at room temperature, the absorbance was measured at 420 nm against a blank, using a UV–vis spectrophotometer. In order to calculate the TFC values of the samples, a four-point calibration curve (y = 0.0559x + 0.0084; R^2^ = 0.999), using the quercetin standard from 10 to 500 μg/mL, was constructed. The results, expressed as the mean value of three replicates ± standard deviation (SD), were reported as micrograms of quercetin equivalents (μg QE) per mL of the sample.

### 3.13. Analyses of Biophenols by HPLC-DAD

The HPLC analyses of the biocompounds were conducted using a Shimadzu 2010 HPLC system (SPD-M20A, Shimadzu, Kyoto, Japan) equipped with a UV–vis DAD, a binary rapid separation pump (LC-20A, Shimadzu, Kyoto, Japan), and an autosampler (SIL-20A, Shimadzu, Kyoto, Japan). The separation was conducted using a Mediterranea SEA C18 reverse-phase chromatography column (4.6 mm i.d. × 25 cm; 5 μm). The gradient method that was chosen following a series of preliminary studies used a mixture of 0.1% formic acid grade water (mobile phase A) and acetonitrile (mobile phase B). The total runtime of the method was 51 min, and the concentration gradient was varied as follows: initially 10% B, at 20 min 22% B, at 40 min 40% B, at 45 min 10% B, and at 51 min 10% B. A constant flow rate of 0.6 mL/min, room temperature, and an injection volume of 10 μL were used [79]. The identification of the compounds was performed by overlapping the chromatograms of the samples and the standard ones under the same chromatographic conditions. Quantification was performed by constructing calibration curves for each standard phenolic, which was analyzed at four concentrations ranging from 0.1 to 1.0 mg/mL of DMSO.

### 3.14. Antioxidant Activity of AM, AMK, and AWK

#### 3.14.1. DPPH

The free radical scavenging capacity of all compounds was determined by DPPH assay, according to a known protocol [80]. Briefly, 100 μL samples (at concentrations of 10, 5, and 1 mg per mL of DMSO) were blended with 100 μL of DPPH (0.1 mM in MeOH). Then, MeOH was added to obtain a final volume of 3 mL and mixed. The tubes were kept in the dark for 30 min and the absorbance was read using a UV–vis spectrophotometer (model V-550, Jasco Europe, Cremella (Co), Italy) at 517 nm against a blank (3 mL of MeOH). The control was prepared by mixing 2.8 mL of MeOH, 100 μL of DPPH 0.1 mM, and 100 μL DMSO. The standard curve was prepared using different concentrations of Trolox (as the standard solution for calibration), and the results were expressed as µg of Trolox Equivalents (TE) per mL of the sample. The EC_50_ values, determined by GraphPad Prism 10.3.1 software (GraphPad Inc., San Diego, CA, USA), were also reported.

#### 3.14.2. ABTS

The ABTS assay measures the ability of antioxidant compounds to scavenge the ABTS (2,2′-azino-bis (3-ethylbenzothiazoline-6-sulfonic acid)) generated in an aqueous phase, compared with Trolox as the standard. The total antioxidant activity of kefir samples was evaluated using an ABTS^+^ radical cation decolorization assay [81]. ABTS^+^ cation radical was generated by the reaction between ABTS (7 mM in water) and potassium persulfate (2.45 mM) (1:1 *v/v*), stored in the dark at ambient temperature for 12–16 h before use. ABTS^+^ stock solution was diluted with ethanol to obtain a working solution with an absorbance of 0.700 at the wavelength of 734 nm. Then, 100 µL of each sample (at concentrations of 1.0, 5, 1 mg per mL of DMSO) was added to 2.9 mL of diluted ABTS^+^ working solution and the absorbance was measured at 734 nm after 5 min of incubation at room temperature in the dark against a control prepared by mixing 2.9 mL of EtOH with 100 µL of DMSO. A standard curve was plotted, recording the absorbance of different concentrations of Trolox, and the results were reported as µg of Trolox Equivalents (TE) per mL of the sample. The EC_50_ values, determined by GraphPad Prism 10.3.1 software (GraphPad Inc., San Diego, CA, USA), were also reported.

#### 3.14.3. FRAP Assay

This method is based upon the reduction of Fe^3+^-TPTZ (ferric tripyridyl triazine) to Fe^2+^-TPTZ by the antioxidants. The reduction of Fe^3+^ results in the appearance of a blue color, which is quantified by measuring the absorbance at a wavelength of 593 nm. The working solution (FRAP reagent) was prepared by blending 10 volumes of sodium acetate buffer (0.25 M, pH 3.6), 1 volume of TPTZ (10 mM in 40 mM HCl), and 1 volume of FeCl_3_ (20 mM). In a test tube, 100 µL of the sample prepared by dissolving 100 mg of freeze-dried milk in 1 mL of DMSO, 900 µL of water, and 2 mL of the FRAP reagent were mixed, stored at room temperature for 10 min, and the absorbance was read. The antioxidant capacity of the tested solutions was calculated by constructing the calibration curve of the standard FeSO_4_ (1 mM) [82]. The FRAP assay results were reported as µg of Fe^2+^ per mL of sample.

### 3.15. Rheological Analyses

The rheological measurements of all samples were carried out by a rheometer SR 5000 (Rheometrics, Piscataway, NJ, USA), using a plate geometry with a diameter of 25 mm. This instrument worked in controlled stress or controlled shear and was connected to a temperature control system, printer, plotter, and computerized data acquisition system. Each experiment had a test temperature controlled by means of the control system, with a concentration of 3 wt% carefully loaded into the cup in which the fixture was slowly lowered. Rheological measurements were subsequently started after 3–5 min to allow for temperature equilibration. The gap was set at 2 mm and a temperature of 25 °C was utilized for rheological determinations. The rheological response of the different samples was modeled using the RSI ORCHESTRATOR software 7.2 provided by Rheometer Scientific (Piscataway, NJ, USA). The apparent viscosity values were calculated at a shear rate ranging from 1 to 50 s^−1^ and were expressed as Pa·s [83,84].

### 3.16. SEM

Morphological features of samples were examined using a Phenom ProX scanning electron microscope (Thermo Fisher Scientific, Hillsboro, OR, USA). Before analysis, a thin layer of carbon (approximately 5 nm) was deposited onto the sample surface, using a carbon coater to improve conductivity. Both secondary electron (SE) and backscattered electron (BSE) imaging techniques were employed to visualize the surface morphology and crystal structure of the samples. The SEM was operated at an accelerating voltage of 5 kV [85].

### 3.17. Evaluation of Pearson Correlation Coefficients

The Pearson correlation measures the strength of the linear relationship between two variables. It takes values between −1 and 1, where 1, 0, and −1 indicate a perfect match, no correlation, and perfect negative correlation, respectively. It has a value between −1 to 1, with a value of −1 meaning a total negative linear correlation, 0 being no correlation, and +1 meaning a total positive correlation [86]. Pearson correlation coefficients (PMCC) between the TPC, TFC, ABTS, DPPH, and FRAP of each sample were calculated using GraphPad Prism 10.3.1 software (GraphPad Software, San Diego, CA, USA).

### 3.18. Statistical Analyses

The experiments were conducted in three replications and the analyses’ averages were subjected to variance analysis using GraphPad Prism 10.3.1 software (GraphPad Software, San Diego, CA, USA). This study opted for one-way ANOVA followed by Dunnett’s test to determine the differences between the means ± standard deviations of pH and titratable alkalinity and Tukey’s test for evaluating the significance of protein content and FRAP values. Two-way ANOVA, followed by Sidak’s test, was used to calculate the significant differences in grain growth, whereas Tukey’s test was applied to make multiple comparisons for fatty acid, flavonoid, phenolic content, and EC_50_ (DPPH and ABTS). Significance was established at *p* values < 0.05 (*), *p* < 0.01 (**), *p* < 0.001 (***), and *p* < 0.0001 (****).

## 4. Conclusions

In recent years, there has been an increased need to develop dairy substitutes for the sake of physical health. Based on research findings about the fermentation of plant-based milk alternatives [1,2,3,4,5,6], it can be concluded that they provided a good alternative as a probiotic carrier. The type of kefir grain and vegetable substrate played a fundamental role in determining which compounds were metabolized and produced during fermentation.

The present study was designed to determine the effect of milk and water kefir grains on the physicochemical, microbiological, and antioxidant properties of almond milk kefir samples. The findings provided further evidence that the type of kefir culture differentially affected the nutritional profile, biological activity, and rheological and morphological features of the final fermented beverages. First of all, the microbiological count showed that the bacteria and yeast population in both AMK and AWK exceeded the legally required minimum levels of probiotic bacteria (6–7 log_10_ cfu/g). The protein content was higher in the AMK than in the AWK, which was consequently more digestible. The total phenol and flavonoid content of the AMK was also higher than that of the AWK, although the antioxidant capacity of both fermented beverages, as measured by ABTS and DPPH, was higher than almond milk, they did not differ significantly. Both fermented beverages highlighted an increment in monounsaturated fatty acids and a reduction in saturated fatty acids compared with almond milk. Only the AWK was enriched with α-methyl linoleate after fermentation. Morphological characterizations showed a higher porosity in the AWK than in the AMK, but both fermented beverages showed a non-Newtonian pseudoplastic behavior at a low shear rate. Therefore, further studies are required to improve nutritional values and the functional properties of fermented almond beverages, as well as sensory analyses, to establish the best beverage quality depending on the starter cultures.

## Figures and Tables

**Table 1:** Water content (%) of AM, AMK, and AWK.

Samples	H_2_O (%)
AM	90.92 ± 1.00 ^a^
AMK	82.45 ± 1.00 ^c^
AWK	87.22 ± 1.00 ^b^

Values were expressed as the mean ± standard deviation. Letters indicated the significance calculated by one-way ANOVA with Tukey’s test. Values with different letters were significantly different.

**Table 2:** Microbial counts expressed as log_10_ cfu/g carried out on kefir and grains after fermentation.

Microorganism	MKGs	WKGs	AMK	AWK
Total bacteria	4.41 ± 0.01 ^b^	5.39 ± 0.01^a^	4.14 ± 0.01 ^a^	3.11 ± 0.01 ^b^
LAB	3.14 ± 0.02 ^a^	2.91 ± 0.10 ^b^	0 ^c^	0 ^c^
Lactococci	4.57 ± 0.01 ^a^	3.49 ± 0.01 ^b^	4.60 ± 0.01 ^a^	3.48 ± 0.01 ^b^
AAB	3.55 ± 0.03 ^b^	4.30 ± 0.03 ^a^	2.55 ± 0.01 ^a^	2.29 ± 0.01 ^b^
Total yeast	4.47 ± 0.01 ^a^	2.30 ± 0.01 ^b^	4.43 ± 0.03 ^a^	1.30 ± 0.01 ^b^

Values were given as the mean ± standard deviation of three replications. Letters indicated significance within the same microbial group between MKGs vs. WKGs and the AMK vs. AWK after 24 h of fermentation, calculated by using two-way ANOVA with Sidak’s test. Values with the same letter were not significantly different (ns), while values with different letters were significantly different.

**Table 3:** pH values and total acidity of almond milk and fermented beverages.

Samples	pH	Total Acidity (%)
AM	7.68 ± 0.16 ^a^	0.03 ± 0.01 ^c^
AMK	4.71 ± 0.02 ^b^	0.11 ± 0.04 ^b^
AWK	4.30 ± 0.03 ^c^	0.21 ± 0.01 ^a^

Values were expressed as the mean ± standard deviation. Letters indicated the significance within the same column calculated by one-way ANOVA with Dunnett’s test. Values with the same letter were not significantly different (ns), while values with different letters were significantly different.

**Table 4:** Total protein content (mg/100 mL).

Samples	Protein
AM	0.40 ± 0.01 ^c^
AMK	1.07 ± 0.12 ^a^
AWK	0.58 ± 0.15 ^b^

Values were expressed as the mean ± standard deviation. Letters indicate significance within the same column calculated by one-way ANOVA with Tukey’s test. Values with different letters were significantly different.

**Table 5:** Fatty acid content of AM and kefir samples, expressed as mg/g.

Fatty Acid	AM	AMK	AWK
C4:0	120.83 ± 2.44 ^a^	63.27 ± 2.32 ^b^	77.67 ± 0.40 ^b^
C12:0	8.16 ± 0.21 ^a^	9.28 ± 0.19 ^a^	5.39 ± 0.03 ^b^
C14:0	2.34 ± 0.01 ^a^	2.79 ± 0.01 ^a^	2.55 ± 0.01 ^a^
C16:0	12.52 ± 0.07 ^b^	3.55 ± 0.04 ^c^	20.75 ± 1.12 ^a^
C17:0	0 ^c^	17.02 ± 0.03 ^a^	17.08 ± 0.01 ^a^
C18:0	12.22 ± 0.08 ^b^	15.38 ± 0.26 ^a^	16.18 ± 0.36 ^a^
C18:1n-9	188.77 ± 2.20 ^b^	278.71 ± 0.97 ^a^	276.63 ± 10.63 ^a^
C16:1n-7	52.59 ± 0.01 ^a^	52.60 ± 0.01 ^a^	52.60 ± 0.01 ^a^
C18:2n-6	34.37 ± 0.48 ^c^	48.91 ± 0.68 ^b^	71.32 ± 2.69 ^a^
C18:3n-3	0 ^b^	0 ^b^	11.62 ± 0.01 ^a^
C20:4n-6	15.51 ± 0.01 ^a^	15.54 ± 0.01 ^a^	15.53 ± 0.01 ^a^
SFA	156.07 ± 2.81	111.29 ± 2.85	138.62 ± 1.93
MUFA	241.36 ± 2.21	331.31 ± 0.98	329.23 ± 10.64
PUFA	49.88 ± 0.49	64.45 ± 0.69	98.47 ± 2.71

Results were given as the means ± standard deviations of triplicate analyses of each sample. SFA = saturated fatty acid, MUFA = monounsaturated fatty acid, n-9 = Omega-9, PUFAs = polyunsaturated fatty acids, n-6 = Omega-6 fatty acids, and n-3 = Omega-3 fatty acids. Letters indicated the significance of each fatty acid between the AM, AMK, and AWK, calculated by two-way ANOVA with Tukey’s test.

**Table 6:** Total phenolic and flavonoid content of AM, AMK, and AWK.

Samples	TPC µg GAE/mL	TFC µg QE/mL
AM	165.43 ± 0.57 ^c^	7.72 ± 0.30 ^c^
AMK	365.81 ± 4.75 ^a^	31.64 ± 0.57 ^a^
AWK	275.98 ± 1.86 ^b^	14.56 ± 0.39 ^b^

Values were expressed as the mean ± standard deviation of three replications. Letters indicated the significance within the same column calculated by two-way ANOVA with Tukey’s test. Values with different letters were significantly different.

**Table 7:** Identification and quantification of biophenolics in AM, AMK, and AWK. Values were given as µg/g and represented averages (± SD) of triplicate measurements.

Compounds	AM	AMK	AWK
Gallic acid	n.d.	1.35 ± 0.01	1.32 ± 0.01
Chlorogenic acid	n.d.	2.46 ± 0.01	2.47 ± 0.01
Vanillic acid	6.76 ± 0.31	6.99 ± 0.01	6.48 ± 0.01
Quercetin	n.d.	8.82 ± 0.01	8.82 ± 0.01
Catechin	6.40 ± 0.03	7.89± 0.02	6.36 ± 0.02
*p*-coumaric acid	5.83 ± 1.45	6.43 ± 0.01	10.81 ± 0.15
Ellagic acid	n.d.	0.10 ± 0.01	0.23 ± 0.01
Ferulic acid	n.d.	0.32 ± 0.02	2.87 ± 0.14
Caffeic acid	n.d.	n.d.	n.d.
Rutin	n.d.	n.d.	n.d.
EGCG	n.d.	n.d.	n.d.

**Table 8:** Trolox equivalent antioxidant capacity (TEAC) values, expressed as µg TE/mL, against DPPH, and EC_50_ values, expressed as µg/mL, of AM, AMK, and AWK.

Samples	Concentration			EC_50_
	333.33 µg/mL	166.67 µg/mL	33.33 µg/mL	(µg/mL)
AM	221.35 ± 1.51	206.71 ± 1.01	182.78± 5.55	11.36 ± 1.05 ^a^
AMK	231.36 ± 0.50	223.85 ± 5.05	184.21 ± 5.55	4.68 ± 0.66 ^b^
AWK	232.78 ± 1.51	224.93 ± 6.56	198.50 ± 2.52	3.91 ± 0.58 ^b^

EC_50_ values were reported as the average ± standard deviations of three replicates. Values with the same letter were not significantly different, values with different letters were significantly different. The significance was calculated by two-way ANOVA with Tukey’s test.

**Table 9:** Trolox equivalent antioxidant capacity (TEAC) values, expressed as µg TE/mL, against ABTS•^+^ and EC_50_ values, expressed as µg/mL, of AM, AMK, and AWK.

Samples	Concentration			EC_50_
	333.33 µg/mL	166.67 µg/mL	33.33 µg/mL	(µg/mL)
AM	5.67 ± 0.07	5.50 ± 0.01	5.24± 0.04	30.87 ± 1.49 ^b^
AMK	6.21 ± 0.04	5.38 ± 0.05	4.82 ± 0.52	13.02 ± 1.11 ^a^
AWK	5.77 ± 0.01	5.33 ± 0.02	4.82 ± 0.52	17.94 ± 1.24 ^a^

EC_50_ values were reported as the average ± standard deviations of three replicates. Values with the same letter were not significantly different, values with different letters were significantly different. The significance was calculated by two-way ANOVA with Tukey’s test.

**Table 10:** FRAP values (μM/mL FeSO_4_) of AM, AMK, and AWK.

Samples	µM/mL FeSO_4_
BHT	145.81 ± 0.03 ^c^
AM	98.96 ± 0.01 ^d^
AMK	157.75 ± 0.05 ^b^
AWK	500.09 ± 1.93 ^a^

Values were reported as the average ± standard deviations of three replicates. Values with the same letter were not significantly different, values with different letters were significantly different. The significance was calculated by one-way ANOVA with Tukey’s test.

**Table 11:** Values of apparent viscosity (Pa·s) as a function of shear rate (s^−1^) of AM, AMK, and AWK.

Shear Rate (s^−1^)	AM	AMG	AWG
1.01	0.577	0.629	0.706
1.26	0.419	0.439	0.471
1.586	0.355	0.362	0.375
1.20	0.318	0.323	0.331
2.51	0.296	0.301	0.305
5.012	0.270	0.272	0.273
7.94	0.265	0.266	0.267
9.99	0.264	0.265	0.266
12.58	0.264	0.265	0.265
25.12	0.267	0.267	0.267
39.81	0.270	0.270	0.271
50.12	0.273	0.272	0.273

**Table 12:** Pearson correlations between antioxidant capacity parameters, phenolic, and flavonoid content in kefir AM, AMK, and AWK.

AM	TPC	TFC	FRAP	DPPH	ABTS
**TPC**		+(r: 0.99)	−(r: −0.98)	−(r: −0.5)	+(r: 0.86)
**TFC**	+(r: 0.73)		−(r: −0.99)	−(r: −0.39)	+(r: 0.80)
**FRAP**	−(r: −0.98)	−(r: −0.99)		+(r: 0.32)	−(r: −0.75)
**DPPH**	−(r: −0.5)	−(r: −0.39)	+(r: 0.32)		−(r: 0.86)
**ABTS**	+(r: 0.86)	+(r: 0.79)	−(r: −0.75)	−(r: 0.86)	
**AMK**	**TPC**	**TFC**	**FRAP**	**DPPH**	**ABTS**
**TPC**		+(r: 0.72)	−(r: −0.82)	+(r: 1)	+(r: 1)
**TFC**	+(r: 0.72)		−(r: −0.98)	+(r: −0.73)	+(r: −0.73)
**FRAP**	−(r: −0.82)	−(r: −0.98)		−(r: −0.81)	−(r: −0.81)
**DPPH**	+(r: 1) **	+(r: −0.73)	−(r: −0.81)		+(r: 1)
**ABTS**	+(r: 1)	+(r: −0.73)	−(r: −0.81)	+(r: 1)	
**AWK**	**TPC**	**TFC**	**FRAP**	**DPPH**	**ABTS**
**TPC**		+(r: 0.99)	−(r: −0.96)	+(r: 0.86)	+(r: 0.86)
**TFC**	+(r: 0.99)		−(r: 0.95)	+(r: 0.87)	+(r: 0.87)
**FRAP**	−(r: −0.96)	−(r: 0.95)		−(r: 0.68)	−(r: 0.68)
**DPPH**	+(r: 0.86)	+(r: 0.87)	−(r: 0.68)		Perfect line
**ABTS**	+(r: 0.86)	+(r: 0.87)	−(r: 0.68)	Perfect line	

## Data Availability

The data presented in this study are available on request from the corresponding author.
